# Identification of optimal reference genes for gene expression normalization in human osteosarcoma cell lines under proliferative conditions

**DOI:** 10.3389/fgene.2022.989990

**Published:** 2022-12-09

**Authors:** Xiaoming Dong, Qiwei Yang, Zhenwu Du, Guizhen Zhang, Chuankai Shi, Xuyuan Qin, Yang Song

**Affiliations:** ^1^ Medical Center of Orthopaedics, The Second Hospital of Jilin University, Changchun, China; ^2^ Gene Testing Center of Changchun Tumor Hospital, Changchun, China

**Keywords:** reverse transcription quantitative polymerase chain reaction, reference gene, human osteosarcoma, normalization, gene expression

## Abstract

The molecular pathogenesis and therapeutic target research studies on osteosarcoma (OS) have developed well during the last few years using various OS cell lines with reverse transcription quantitative polymerase chain reaction (RT-qPCR). However, the identification of suitable reference genes of RT-qPCR for OS cell lines has not been reported. Here, we conducted the normalization research of 12 reference genes (GAPDH, ACTB, 18S, B2M, ALAS1, GUSB, HPRT1, HMBS, PPIA, PUM1, RPL29, and TBP) for gene expression analysis in four kinds of human OS cell lines (U2OS, Saos-2, HOS, and MG-63) to improve the investigation of molecular mechanisms and the accuracy of diagnosis and prognostic molecular targets of OS. The gene expression stability and applicability of the 12 reference gene candidates were determined using geNorm, NormFinder, and BestKeeper software. The results indicated that PUM1 and the combination of PPIA + ALAS1 were recommended as the optimal reference gene in these four different sources of human OS cell lines under proliferative conditions. The present study identified the most suitable reference genes and reference gene combinations for OS cell lines under proliferative conditions in order to use in gene expression profile analysis. A reliable standardized method has the potential to improve the understanding of the biological mechanisms underlying OS in the future.

## Introduction

Osteosarcoma (OS) is an aggressive and mesenchymal stem/stromal cells tumor that mainly affects children, adolescents, and young adults (Czarnecka et al., 2020). Over the past decades, the therapeutic management of OS has remained largely insufficient, and patient survival has not improved (Corre et al., 2020). Recently, several genomic studies by whole-genome sequencing (WGS) and/or whole-exome sequencing (WES) explored the pathophysiology and genetics of OS, which identified genetic heterogeneity, including chromosomal abnormalities, mutations, and abnormal genes expression (Bousquet et al., 2006; Ho et al., 2017). The investigation of molecular pathogenesis and therapeutic targets of OS has been developed by various OS cell lines with reverse transcription quantitative polymerase chain reaction (RT-qPCR). However, the screening of appropriate reference genes of RT-qPCR for OS cell lines under proliferative conditions has never been reported.

RT-qPCR is regularly applied in gene expression quantification and is currently considered the gold standard for precise, sensitive, and rapid quantification of gene expression. RT-qPCR as a key method is often applied on the investigation of molecular pathogenesis and therapeutic targets for OS (Yang et al., 2020). Relative quantification is a pivotal and commonly used technique to estimate RT-qPCR data, while the expression levels of target genes are compared to those of a stably expressed endogenous control gene, determined simultaneously in the same biological sample. The reference genes allow quantification to be normalized against innate variation in RNA extraction, integrity, and cDNA synthesis efficiency. Therefore, the gene expression levels require normalization using reference genes in order to obtain reliable data. The identification of appropriate reference genes is an important stage involved in this approach, as described in our previous works (Song et al., 2018; Wang et al., 2017). It is important for the ideal reference genes to be universally valid under experimental conditions (Radonić et al., 2004; Derveaux,et al., 2010). In general, cellular maintenance genes, such as glyceraldehydes-3-phosphate dehydrogenase (GAPDH) (Yang et al., 2020), β-actin (ACTB), and ribosomal RNA (18S rRNA) (Studer et al., 2012), are considered reference genes to evaluate the variability among clinical samples. However, several studies have demonstrated that the expression levels of these regularly applied reference genes vary in distinct tissues or various treatments in the same samples (Song et al., 2016; Yang et al., 2015) and different cell lines or cell types (He et al., 2015).

OS is an aggressive malignant neoplasm that arises from primitive transformed cells of mesenchymal origin, exhibiting osteoblastic differentiation and produces malignant osteoid (Luetke et al., 2014). HOS, MG-63, Saos-2, and U2OS cell lines are the most commonly used OS cell lines in *in vitro* studies. HOS is a cell line derived from a 13-year-old Caucasian girl. It is sensitive to both virus and chemical transformation and exhibits a flat morphology, low saturation density, and low plating efficiency in soft agar. MG-63 is a cell line derived from a 14-year-old Caucasian boy and expresses TGF-β receptors I and II (Billiau et al., 1977). Saos-2 is a cell line derived from the primary OS of an 11-year-old Caucasian girl who had been treated by radiotherapy and various drug therapies (Fogh et al., 1977). Saos-2 cells can be fully differentiated in a manner that the osteoblastic cells naturally do (Hausser and Brenner, 2005). Also, it is a valuable model for studying events associated with the late osteoblastic differentiation stage in human cells (Marino S, et al., 2016). The cells cannot become tumorigenic in immunosuppressed mice, but they can form colonies in a semi-solid medium. The cells express EGFR and TGF-β receptor I and II (Karnieli et al., 1996; Morris et al., 1996; Werner et al., 1996). U2OS cell line was derived from a tibial moderately differentiated sarcoma of a 15-year-old Caucasian girl. The cells express insulin-like growth factor I, II receptor, and OS-derived growth factor (Landers et al., 1997; Niforou et al., 2008). Although they are all derived from OS cells, they differ in many aspects, in particular, concerning their proliferation kinetics and the osteoid production (Luetke et al., 2014; Pautke et al., 2004; Grigolo et al., 1998; Ohl et al., 2005; Declercq et al., 2004). Thus, a review of the normalization standards used in the quantitative gene expression studies of human OS cell lines is necessary. To the best of our knowledge, there is no systematic study that has been performed on the selection of suitable reference genes for investigating target gene profiling in these four different sources of human OS cell lines under proliferative conditions.

Our present study aims to identify the most suitable reference genes or set of genes for target gene profiling of OS cell lines under proliferative conditions. The panel stability of 12 common reference genes in four different sources of human OS cell lines was validated. The 12 candidate genes: ACTB, ALAS1, GAPDH, TBP, HPRT1, RPL-29, PBGD, PPIA, PUM1, GUSB, B2M, and 18S rRNA are frequently used as endogenous controls in the context of, but not restricted to, OS. A number of these genes have been identified as optimal reference genes in certain other cancer types, including HPRT1 and ACTB (Huan et al., 2012; Huang et al., 2015). To investigate these genes, three common software packages, geNorm (Vandesompele et al., 2002), NormFinder (Andersen et al., 2004), and BestKeeper (Pfaffl et al., 2004) were used. The aim was to provide useful information for the selection of suitable reference genes in further gene expression studies on OS.

## Materials and methods

### OS cell lines and culture

HOS, Saos-2, MG-63, and U2OS cell lines were purchased from the cell bank of CAS Shanghai Institute (China) and cultivated, according to the recommendation of the supplier, in MEM-EBSS (HyClone, United States) containing 10% FBS (HyClone, United States) and 1% non-essential amino acids (HyClone, United States); McCoy’s 5A media (modified with tricine, HyClone, United States) containing 15% FBS; MEM-EBSS containing 10% FBS and 1% non-essential amino acids; and McCoy’s 5A media (modified with tricine) containing 10% FBS, respectively. All media were supplemented with 100 units of penicillin–streptomycin (HyClone, United States), and all cells were maintained at 37°C in a 5% CO_2_ humidified atmosphere.

### RNA extraction and complementary DNA (cDNA) synthesis

The cell lines were recovered from liquid nitrogen, inoculated into 10 cm^2^ culture dishes, and supplemented the culture medium to 10 ml. The cells were put into the incubator and continued to culture for 24 h before changing the solution, and passaged every 72 h. After two stable passages, the cells were cultured for 72 h. When the cell density reached about 70%, total RNA of each cell was extracted using TRIzol reagent (Invitrogen Life Technologies, United States), following the manufacturer’s protocol. The general steps were as follows: the growth media were removed from the culture dish and washed twice by PBS. 1 ml TRIzol reagent was added directly to the cells in the 10 cm^2^ culture dishes and then harvested into 1.5-ml centrifuge tubes. A total of 0.2 ml of chloroform was added to each tube. Then, the tubes were shaken vigorously by hand for 15 s. The tubes were centrifuged at 12,000 g for 15 min at 4°C. The aqueous phase was removed into new tubes, and 20 U DNase I (Thermo Scientific, United States) was added and incubated at room temperature for 15 min. A total of 0.5 ml of 100% isopropanol was added to the aqueous phase and then centrifuged at 12,000 g for 10 min at 4°C. The supernatant was removed from the tubes, and the pellets were washed twice with 1 ml of 75% ethanol. The RNA pellets were resuspended in RNase-free water. The isolated RNA concentrations and purity were detected using NanoDrop 2000 (Thermo Scientific, United States). The expected purity is OD260/280 between 1.8 and 2.0, while that of OD260/230, above 2.0.

The cDNA synthesis reaction was performed using an All-in-One First-Strand cDNA Synthesis kit (GeneCopoeia, United States) following the manufacturer’s instructions. The total reaction volume was 25 µL. A total of 1 µg total RNA, 1 µL random primer, and RNase-free water were mixed and incubated at 65°C for 10 min and then cooled down immediately on ice. The rest of the reaction reagents were added and incubated at 37°C for 60 min, and the reaction was terminated by heating at 85°C for 5 min. The product cDNA was diluted at a ratio of 1:20 and directly used in RT-qPCR.

### RT-qPCR

RT-qPCR is generally based on the method of our previous works (Wang et al., 2017; Song et al., 2018; Yang et al., 2020). The primers of 12 putative reference genes were selected upon previous studies that are widely used and recognized as good RGs. The primers sequences, product length, and PCR amplification efficiency are shown in Table 1. The primers were synthesized by Sangon Biotech (Shanghai, China). A Roche LightCycler 480 detection system (Roche Diagnostics, Germany) was used for RT-qPCR. Reactions were completed by using All-in-One qPCR mix (GeneCopoeia, United States) following the manufacturer’s instructions. All the samples were run in triplicate. The total reaction volume was 20 μL, containing 2 µL cDNA. The following cycling conditions were used: first, 55°C for 5 min; 95°C for 5 min to pre-denaturation; then, 40 cycles of 95°C for 20 s and 55°C for 20 s; and finally, 72°C for 4 min to fully extend. This cycle was followed by melting curve analysis, and the baseline and cycle threshold values (CP values) were automatically determined for all the plates using Roche LightCycler 480 software (Roche Diagnostics, Germany). There are two methods to verify the specificity of the primers, the RT-qPCR amplification products were detected by 1% agarose gel electrophoresis. A standard curve was constructed for each primer pair to determine the product specificity.

**TABLE 1:** Information of primer sequences for PCR.

Symbol	Official full name	Primer sequence	Product size (bp)	PCR efficiency
18S	18S ribosomal RNA	F:CGGCTACCACATCCAAGGAA	186	1.91
		R:GCTGGAATTACCGCGGCT		
GAPDH	Glyceraldehyde-3-phosphate dehydrogenase	F:GACAGTCAGCCGCATCTTCT	127	1.96
		R:TTAAAAGCAGCCCTGGTGAC		
B2M	Beta-2-microglobulin	F:AGCGTACTCCAAAGATTCAGGTT	306	1.99
		R:ATGATGCTGCTTACATGTCTCGAT		
ACTB	Actin, beta	F:AGAAAATCTGGCACCACACC	173	2.10
		R: TAG​CAC​AGC​CTG​GAT​AGC​AA		
ALAS1	5′-Aminolevulinate synthase 1	F:GGCAGCACAGATGAATCAGA	150	2.00
		R: CCT​CCA​TCG​GTT​TTC​ACA​CT		
GUSB	Glucuronidase, beta	F:AGCCAGTTCCTCATCAATGG	160	1.97
		R: GGT​AGT​GGC​TGG​TAC​GGA​AA		
HPRT1	Hypoxanthine phosphoribosyltransferase 1	F:GACCAGTCAACAGGGGACAT	132	2.06
		R:CCTGACCAAGGAAAGCAAAG		
PBGD	Porphobilinogen deaminase	F:AGTGTGGTGGGAACCAGC	144	1.98
		R:CAGGATGATGGCACTGAACTC		
PPIA	Peptidylprolyl isomerase A	F: AGACAAGGTCCCAAAGAC	118	1.99
		R:ACCACCCTGACACATAAA		
PUM1	Pumilio RNA-binding family member 1	F:CAGGCTGCCTACCAACTCAT	211	2.09
		R:GTTCCCGAACCATCTCATTC		
RPL-29	Ribosomal protein L29	F:GGCGTTGTTGACCCTATTTC	120	2.02
		R: GTG​TGT​GGT​GTG​GTT​CTT​GG		
TBP	TATA box-binding protein	F:TGCACAGGAGCCAAGAGTGAA	132	1.96
		R:CACATCACAGCTCCCCACCA		

The CP values were identified by quantitative comparison of the amplification of the candidate genes. The CP values were calculated to relative quantities (Q) for data analysis according to the equation: Q = 2^−ΔCp^, in view of the PCR efficiencies of the candidate genes that were close enough to two (Livak and Schmittgen, 2001).

### Amplification specificity of primers

**FIGURE 1:**
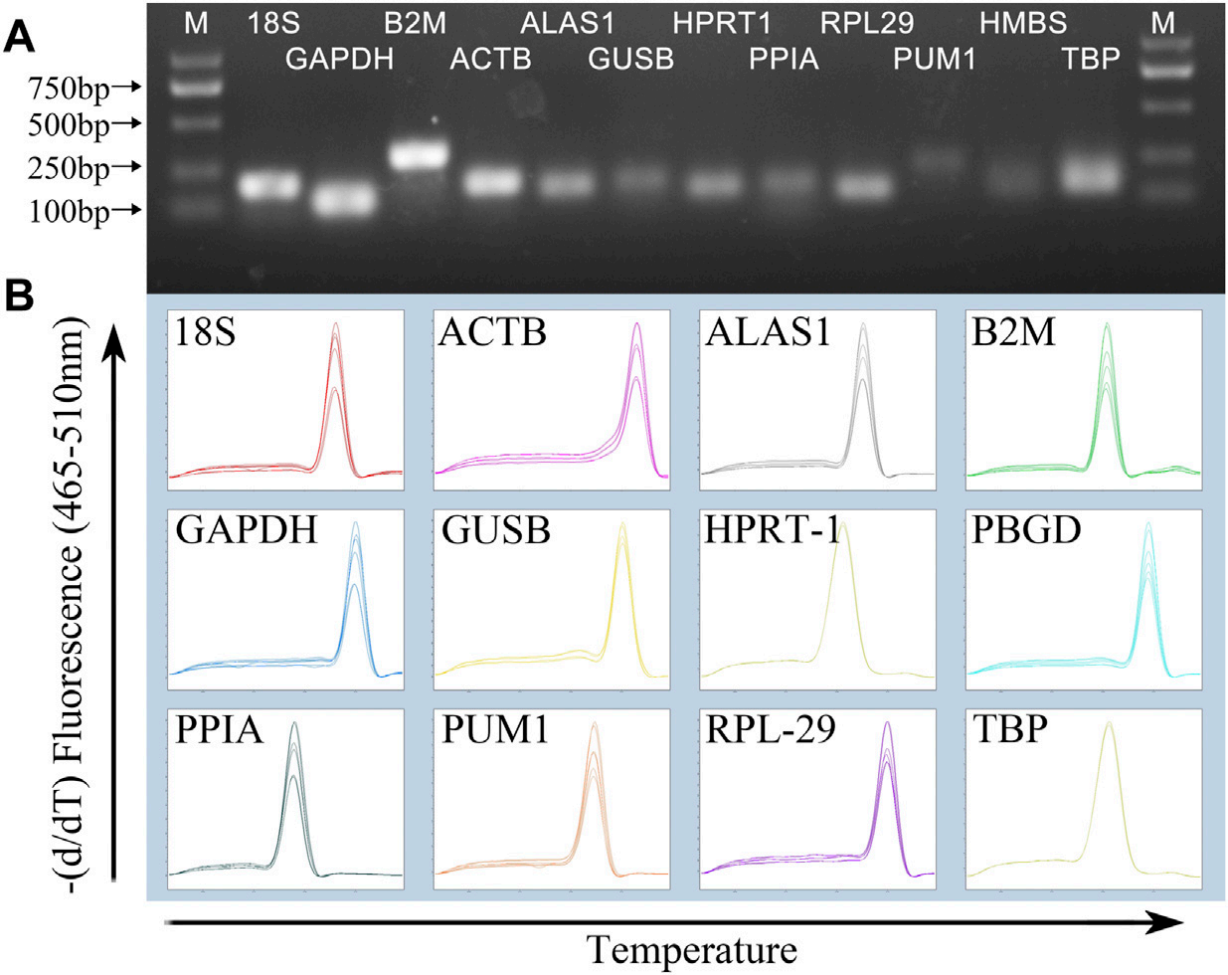
Specificity of RT-qPCR amplification. **(A)** 1% agarose gel electrophoresis of RT-qPCR amplification products and **(B)** melting curve analysis.

Agarose gel electrophoresis image results of PCR products showed that the size of the amplified fragment was consistent with the expected size, with a clear band, and without primer dimers and non-specific bands (Figure 1A). In addition, the melting curve of each gene fragment amplified by qPCR revealed that all curves exhibited a single signal peak (Figure 1B).

### PCR efficiency

A random pool of cDNA from the samples was selected and used for 10-fold serial dilutions, ranging between 0.01X and 10X. The PCR were run in triplicate, as mentioned previously. The PCR efficiency was calculated using the slopes of the calibration curve and by the formula E = 10^−1/slope^. The amplification efficiency range of the reference gene was 1.91–2.10, and all correlation coefficients were >0.99 (Table 1).

### Algorithms and statistical analysis

All the samples were divided into four groups, HOS, Saos-2, MG-63, and U2OS. Algorithms and statistical analysis are also based on the method mentioned in the literature (Song et al., 2018). In order to better evaluate the stability of the reference genes, three frequently used software programs, geNorm (https://genorm.cmgg.be/), NormFinder (http://moma.dk/normfinder-software), and BestKeeper (http://www.gene-quantification.de/bestkeeper.html), were used.

GeNorm is designed to set up reference genes for RT-qPCR and can be used to analyze and determine the M-value, which refers to the stability of the reference gene expression. M-value is defined as the average pairwise variation of a particular reference gene with all other reference genes. Hereas, the pairwise variation was determined as the standard deviation of the logarithmically transformed expression ratios. The default value suggested by geNorm is M = 1.5. The higher the M-value, the less stable and lower the M-value, the more stable the genes are. If M is > 1.5, it is not suitable as a reliable reference gene. GeNorm software can also be used to analyze the pairwise variation value of the normalization factor (V), which has a default value of 0.15. The value of Vn/Vn+1 can be used to determine whether adding a new reference gene affects the normalization factor. If the value of Vn/Vn+1 is > 0.15, it is necessary to use the n + 1 reference genes as internal controls. If it is < 0.15, then it is not necessary to use new reference genes.

NormFinder software is a tool designed to identify the optimal reference gene among a set of candidates, and it has an operation principle similar to geNorm. This program analyzes expression data, ranks the set of candidate normalization genes according to their expression stability, and considers the gene with the minimum expression data as the most stable gene. This software can also be used to compare the stability of inter- and intra-group reference genes and propose an optimal combination of two genes. In this study, we analyzed the data of different biological repeated experiments of the same cell line as one group.

BestKeeper evaluates the candidate reference gene stability based on the standard deviation (SD) and correlation coefficient (r). The genes with SD > 1.00 are suggested to be considered unreliable as a stable control gene, and the remaining genes are ranked according to their r values. The higher the r value, the more the stable and reliable the gene is.

## Results

### Gene expression profiles

**FIGURE 2:**
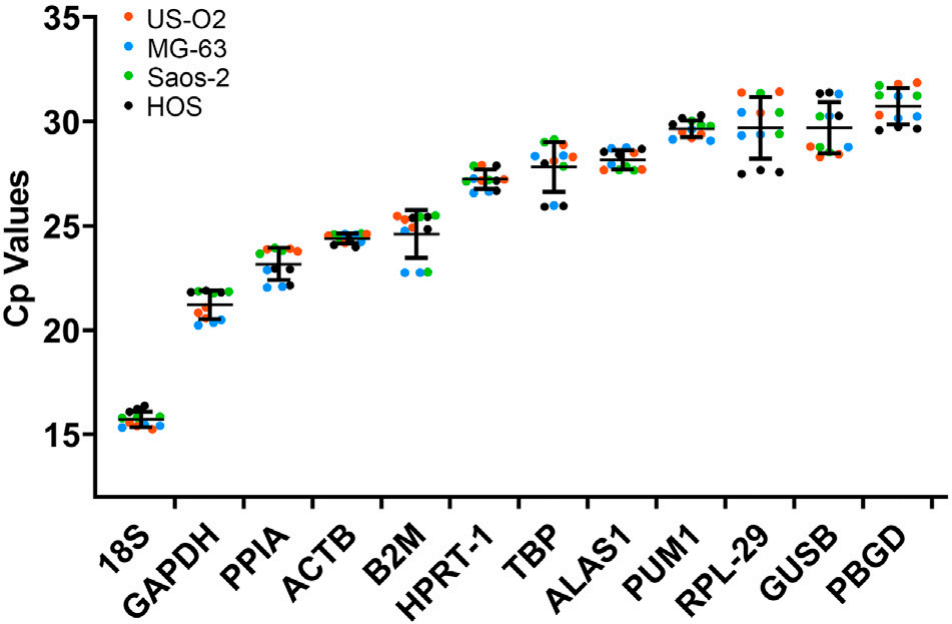
CP values of the reference genes in the experimental samples. Bars represent the mean ± standard deviation.

The expression level of the candidate reference genes was determined by the CP value. As shown in Figure 2, the CP value of all the samples ranged between 15.25 and 31.87. 18S had the smallest CP values of 15.71 ± 0.38, and PBGD had the greatest CP values of 30.73 ± 0.90.

### Stability analysis of the candidate reference gene

**FIGURE 3:**
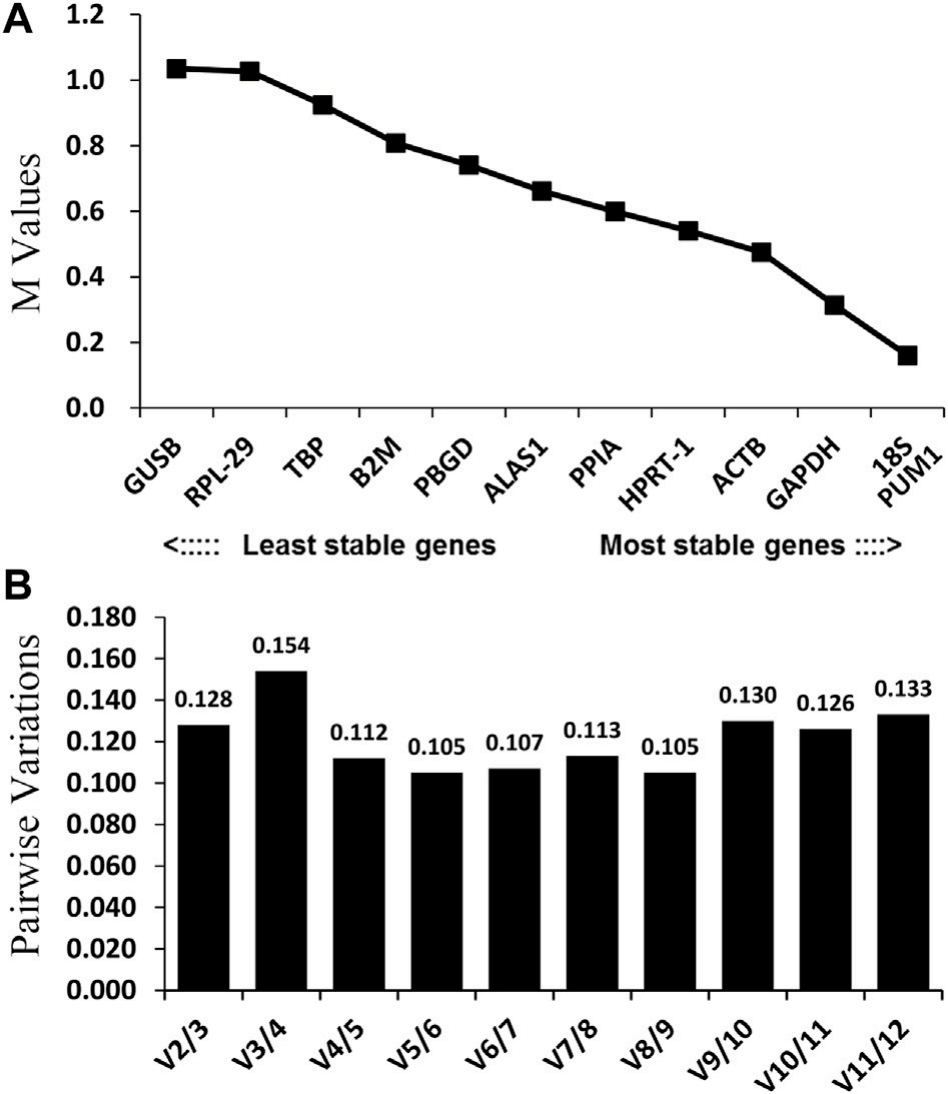
GeNorm analysis of the candidate reference genes. Results are presented according to the output file of the geNorm program. **(A)** Stepwise exclusion of the least stable genes by calculating the M-value. The *x*-axis from left to right indicates the ranking of the reference genes according to their expression stability, and the *y*-axis indicates M; **(B)** determination of the optimal number of reference genes for normalization.

Theoretically, 12 reference genes constitute an appropriate internal for controlling genes. Based on the geNorm program, 18S and PUM1 (Figure 3A; Table 2) have the lowest M-values which indicate that their expression are the most stable expressed reference genes among the four OS cell lines; on the contrary, GUSB and RPL-29 were the most unstable. The data suggest that no real advantage is gained in moving from two reference genes to three (V2/3 = 0.128) (Figure 3B).

**TABLE 2:** Overall ranking of the candidate reference genes’ stability.

Gene	geNorm		NormFinder		BestKeeper		Final ranking	
	M value	Rank	Stability value	Rank	R value	Rank	Geo mean of rank number	Rank
PUM1	0.160	1	0.255	3	0.606	6	2.621	1
PPIA	0.599	6	0.330	5	0.944	1	3.107	2
18S	0.160	1	0.299	4	0.372	8	3.175	3
ACTB	0.475	4	0.207	1	0.053	10	3.420	4
HPRT-1	0.540	5	0.239	2	0.625	5	3.684	5
GAPDH	0.314	3	0.462	7	0.729	4	4.380	6
B2M	0.808	9	0.601	9	0.870	2	5.451	7
ALAS1	0.662	7	0.473	8	0.855	3	5.518	8
PBGD	0.741	8	0.418	6	0.548	7	6.952	9
TBP	0.925	10	0.660	10	0.193	9	9.655	10
RPL-29	1.027	11	0.744	11	—	11	11.000	11
GUSB	1.035	12	0.948	12	—	12	12.000	12

**FIGURE 4:**
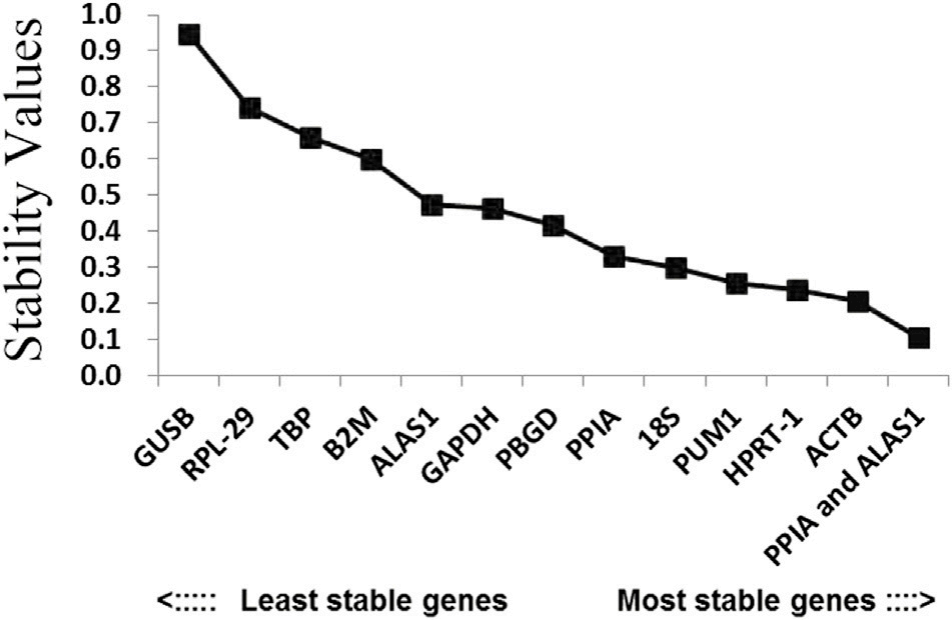
Candidate reference genes for normalization according to their expression stability calculated using the NormFinder program. The *y*-axis represents the stability value. The *x*-axis from left to right represents the ranking of the reference genes.

In order to better evaluate the stability of the 12 reference genes, the present study also used the NormFinder program. As shown in Figure 4 and Table 2, ALAS1 + PPIA were the most stable reference gene combination in this study. ACTB was the most stably expressed gene in this study, followed by HPRT-1. The least two stably expressed gene in the study were GUSB and RPL-29.

**FIGURE 5:**
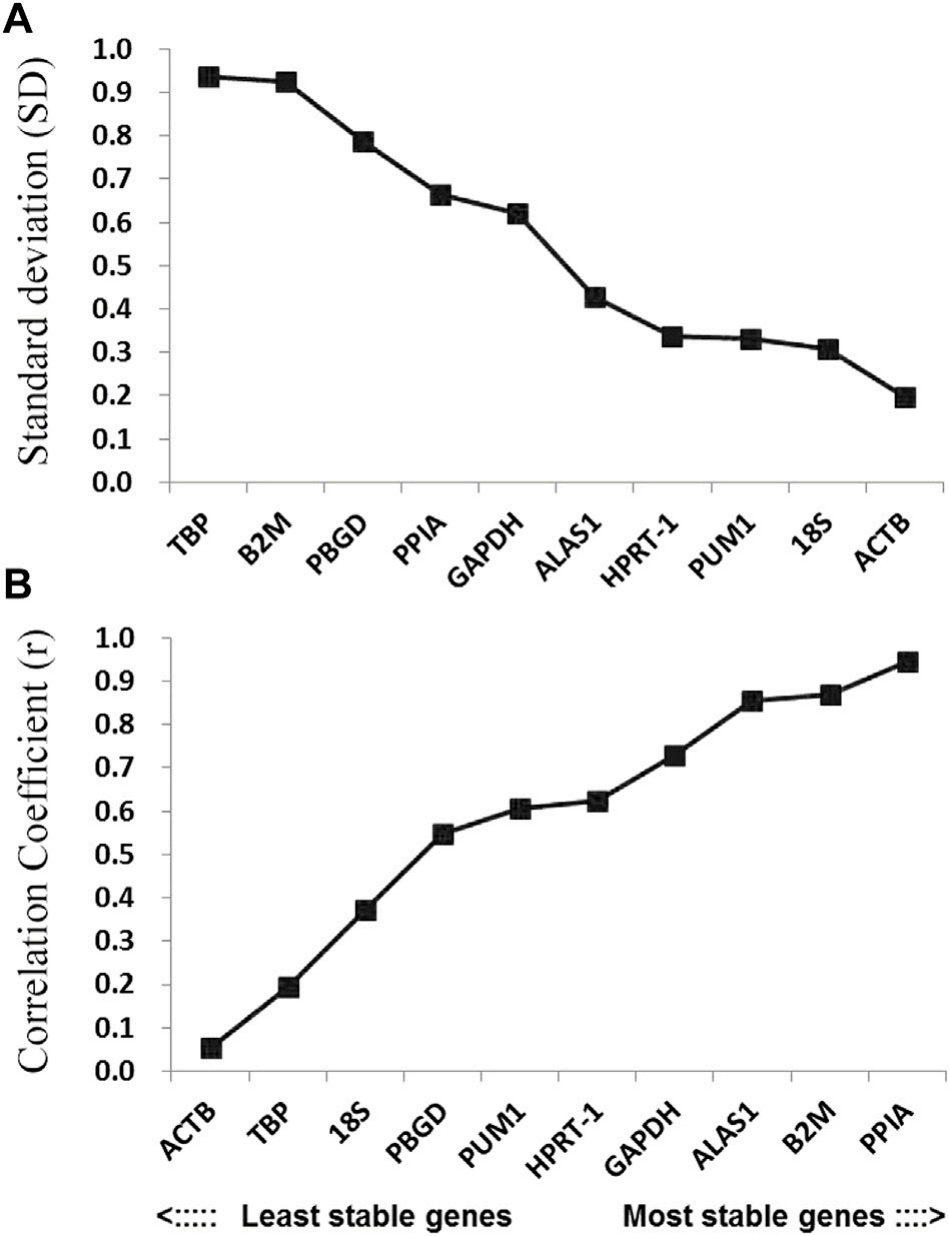
Stability values of the candidate reference genes evaluated using BestKeeper software. **(A)** Standard deviation values of the candidate reference genes; **(B)** coefficient of correlation values of the candidate reference genes.

The BestKeeper program can also be used to compare the stability of internal reference genes. Since the BestKeeper program can only analyze 10 internal reference genes, the two most unstable internal reference genes indicated by the geNorm and NormFinder simultaneous analyses were removed in each group. The BestKeeper analysis demonstrated that the SD values in this study were all <1 (Figure 5A). Considering the r-value, the most stable internal reference gene was PPIA followed by B2M (Figure 5B; Table 2).

### Candidate reference gene ranking

**FIGURE 6:**
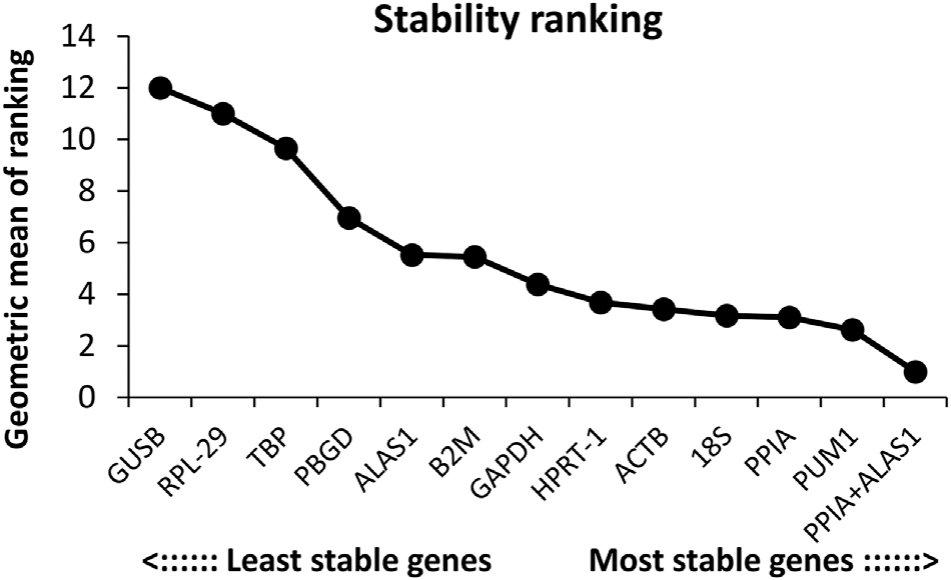
Overall ranking of the candidate reference genes’ stability.

The order of the reference gene stability given by the three software packages was slightly different mainly because they use different algorithms. In order to give the audience a clear suggestion, we applied the method described by Chen et al. (2011). Specifically, we ranked each candidate gene according to the scoring rule of each software, with one being the most stable and 12 being the least stable. In this way, each candidate gene has three rank numbers. Then, we calculated the geometric mean of the three rank numbers of each candidate gene and ranked them accordingly. Taking PUM1 as an example, its rank number in geNorm is 1, in NormFinder is 3, and in BestKeeper is 6; the geometric mean of 1, 3, and 6 is 2.621. The lower geometric mean the candidate gene has, the more stably the candidate gene was expressed. The final ranking of the candidate reference genes expression stability was PUM1 > PPIA >18S > ACTB > HPRT-1 > GAPDH > B2M > ALAS1 > PBGD > TBP > RPL-29 > GUSB (Figure 6; Table 2).

## Discussion

In the present experiment, we investigated the stability of 12 reference genes for mRNA quantification in four human OS cell lines, including HOS, MG-63, Saos-2, and U2OS, and identified stable and reliable internal control genes that may be used in studies examining gene expression by RT-qPCR in OS cells. The cell research techniques based on cell lines can provide more biologically meaningful information than simplified biochemical assays. The most important reasons for their universal adoption are lower operational costs and the ease of operation in terms of preparing and observing the cells. Furthermore, they represent an unlimited self-replicating source that can be grown in almost infinite quantities, yielding unlimited amounts of DNA/RNA that enables studies related to validation and downstream functional analysis (Jain et al., 2020).

In the detection of target gene expression, a gene with a steady expression level is required to normalize the data; these are internal reference genes. Previous studies have indicated that the majority of these commonly used internal control genes have flaws. Their expression level varies significantly depending on various experimental conditions, including different cell types and tissues, different stages of cell proliferation and organ development, and *in vitro* culture (Ali et al., 2015; Ma et al., 2015; Song et al., 2016). To the best of our knowledge, the present study is the first to compare the stability of commonly used internal reference genes in different sources of human OS cell lines under proliferative conditions. With the research development of gene profiling of OS, it is necessary to identify stable and reliable internal control genes. In the present study, the reference genes commonly used in the studies of gene expression in OS were used as were those frequently used in studies examining molecular markers in other cancer tissues.

To obtain accurate experimental data and reliable conclusions, a total of 12 types of common reference genes were compared in terms of their expression stability, and the geNorm, NormFinder, and BestKeeper software programs, commonly used to compare stability between reference genes, were selected for data analysis. The geNorm program was used for initial analysis. This software program is based on a pairwise-comparison statistical model. By calculating the values of M and V, the two most stable reference genes and the optimum number of reference gene combination were determined. Following this analysis, the results suggested that in the study of OS, 18S and PUM1 were the most stable reference genes. In addition, by calculating the value of V, the data suggest that no real advantage is gained in moving from two reference genes to three. ACTB and the combination of PPIA and ALAS1 were considered the most stable reference gene and the best reference gene combinations by the NormFinder software program that is based on the analysis of variance as the statistical model. Finally, the BestKeeper program was used for analysis. The results suggested that PPIA was the most stable reference genes followed by B2M and ALAS1. Since the rank of the candidate genes stability was slightly different, it was possibly caused by different calculation algorithms (Chang et al., 2012; Brugè et al., 2011). In order to give the audience a clear suggestion, we used the method described by Chen et al. (2011) to calculate the geometric average of the ranking of each reference gene in the three software and ranked them accordingly. The geometric average ranking suggested that PUM1 was recommended as the optimal reference gene in these four different sources of human OS cell lines under proliferative conditions. Considering the results of NormFinder (optimal combination of two genes, PPIA + ALAS1) and geNorm (the optimal number of reference gene combination is two), the combination of PPIA + ALAS1 was recommended as a good combination of reference genes in these four different sources of human OS cell lines under proliferative conditions.

To sum up, if only one reference gene is used for a normal gene expression level, PUM1 is the most stable gene; if the experiment allows multiple reference genes to be used for a normal gene expression level, then the combination of PPIA + ALAS1 would be a good choice.

## Conclusion

Our study determined the most suitable reference genes and reference gene combinations used in qPCR for gene expression investigation in OS cell lines under proliferative conditions. The result was that the most stable internal reference gene was PUM1, and the most stable combination was PPIA + ALAS1. As we have demonstrated in previous studies, a reliable standardized method is useful for improving the study of the biological mechanisms of OS in the future.

## Data Availability

The original contributions presented in the study are included in the article/Supplementary Material; further inquiries can be directed to the corresponding author.
